# Diffuse Cerebral Air Emboli After an Esophagogastroduodenoscopy One Month Post Left Atrial Ablation for Atrial Fibrillation

**DOI:** 10.7759/cureus.18101

**Published:** 2021-09-19

**Authors:** Kamil Abu-Shaban, Xiaochen Liu, Bruce Siders

**Affiliations:** 1 Radiology, University of Toledo College of Medicine, Toledo, USA; 2 Radiology, University of Toledo Medical Center, Toledo, USA

**Keywords:** atrial fibrillation management, acute cerebral infarction, cerebral air emboli, left atrial ablation, atrio-esophageal fistula

## Abstract

We present a case of a patient who presented to the emergency department with vague abdominal pain one month after undergoing a left atrial ablation procedure for atrial fibrillation. While in the emergency department, the patient started to have episodes of hematemesis. Esophagogastroduodenoscopy (EGD) was performed and the patient become hypotensive and unresponsive after. Imaging confirmed atrioesophageal fistula and widespread cerebral air emboli and diffuse ischemia. Air emboli were likely introduced through the fistula during the EGD.

## Introduction

Atrial fibrillation is a supraventricular tachyarrhythmia (SVT) that is caused by irregular atrial firing from ectopic foci surrounding the pulmonary veins [1]. A definitive treatment for atrial fibrillation includes catheter ablation of these ectopic foci, which effectively cures arrhythmia in most cases. Risk factors for post-ablation recurrence include increased left atrial size, patient age, and renal dysfunction [2]. Some complications of ablation include atrioesophageal fistula, cardiac tamponade, and femoral hematoma at the catheter access site. We present the case of a patient who underwent ablation for atrial fibrillation and presented to the hospital one month later with hematemesis.

## Case presentation

The patient is a 63-year-old male with a history of atrial fibrillation and dilated cardiomyopathy with an ejection fraction (EF) of 15% presented to the emergency department (ED) with nagging, vague abdominal pain, which started early in the afternoon and woke the patient up from sleep. 

Chest x-ray and computed tomography (CT) abdomen and pelvis with contrast were ordered in the emergency department (ED). The imaging showed bibasilar opacities, geographic splenic infarcts, and extensive colonic diverticulosis. In the ED, the patient experienced episodes of hematemesis and was admitted to the intensive care unit (ICU) with continued mental deterioration. A swab from the ED showed a positive coronavirus disease (COVID) test. 

Upon further review, it was discovered that the patient had a recent left atrial radiofrequency ablation one month prior at an outside hospital. Gastroenterology was consulted for gastrointestinal hemorrhage. An esophagogastroduodenoscopy (EGD) was performed, which showed a moderate amount of clots and blood in the stomach with limited visualization. No documented intervention was performed to stop the bleeding as the patient experienced intermittent desaturation during the procedure and became hypotensive at the end of the procedure. Pupils at the time were dilated and unresponsive. 

**Figure 1:**
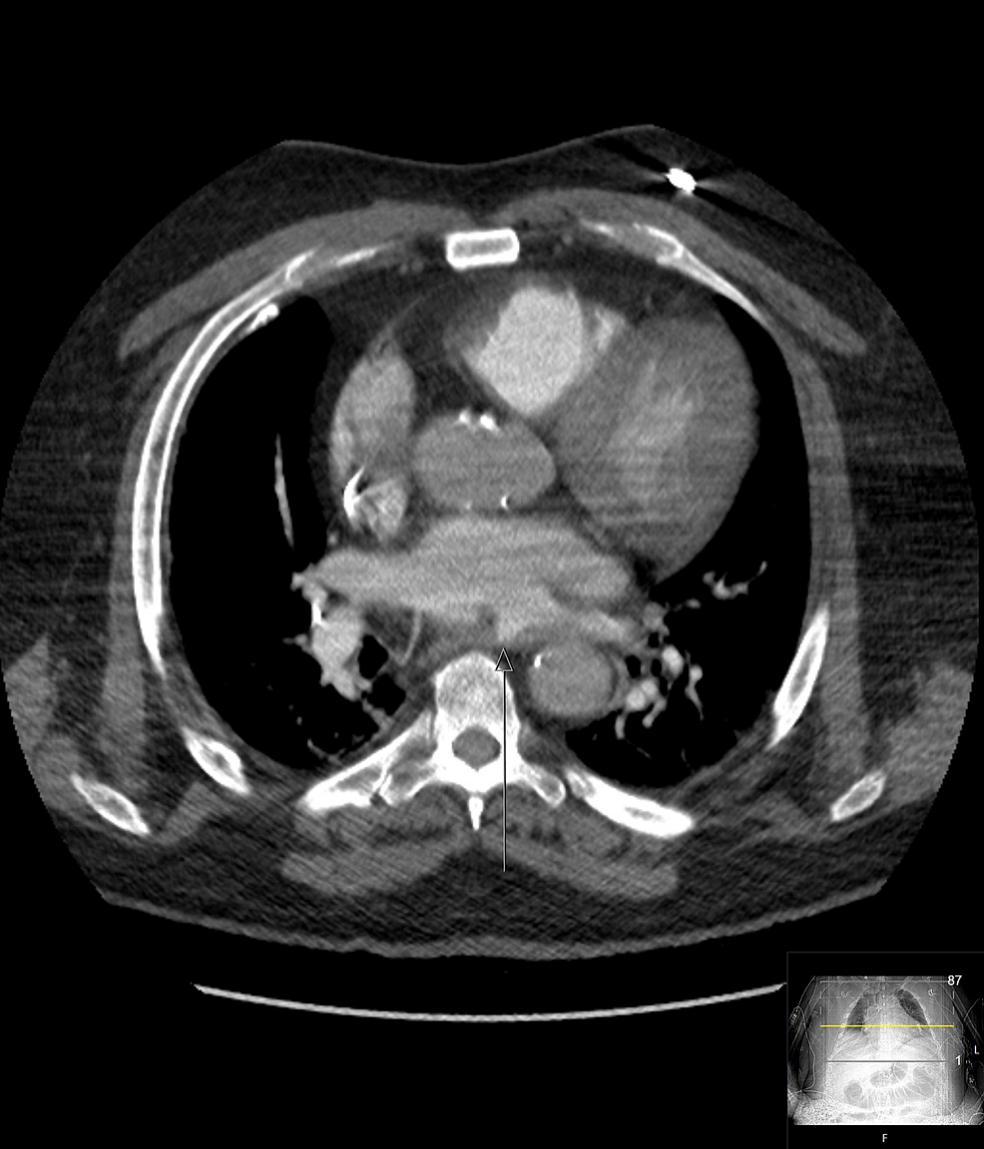
Unusual outpouching of contrast along the posterior aspect of the left atrium directly adjacent to the esophageal lumen. The arrow shows the location of the fistula diagnosed on CT angiography. CT angiography axial chest. CT: computed tomography.

**Figure 2:**
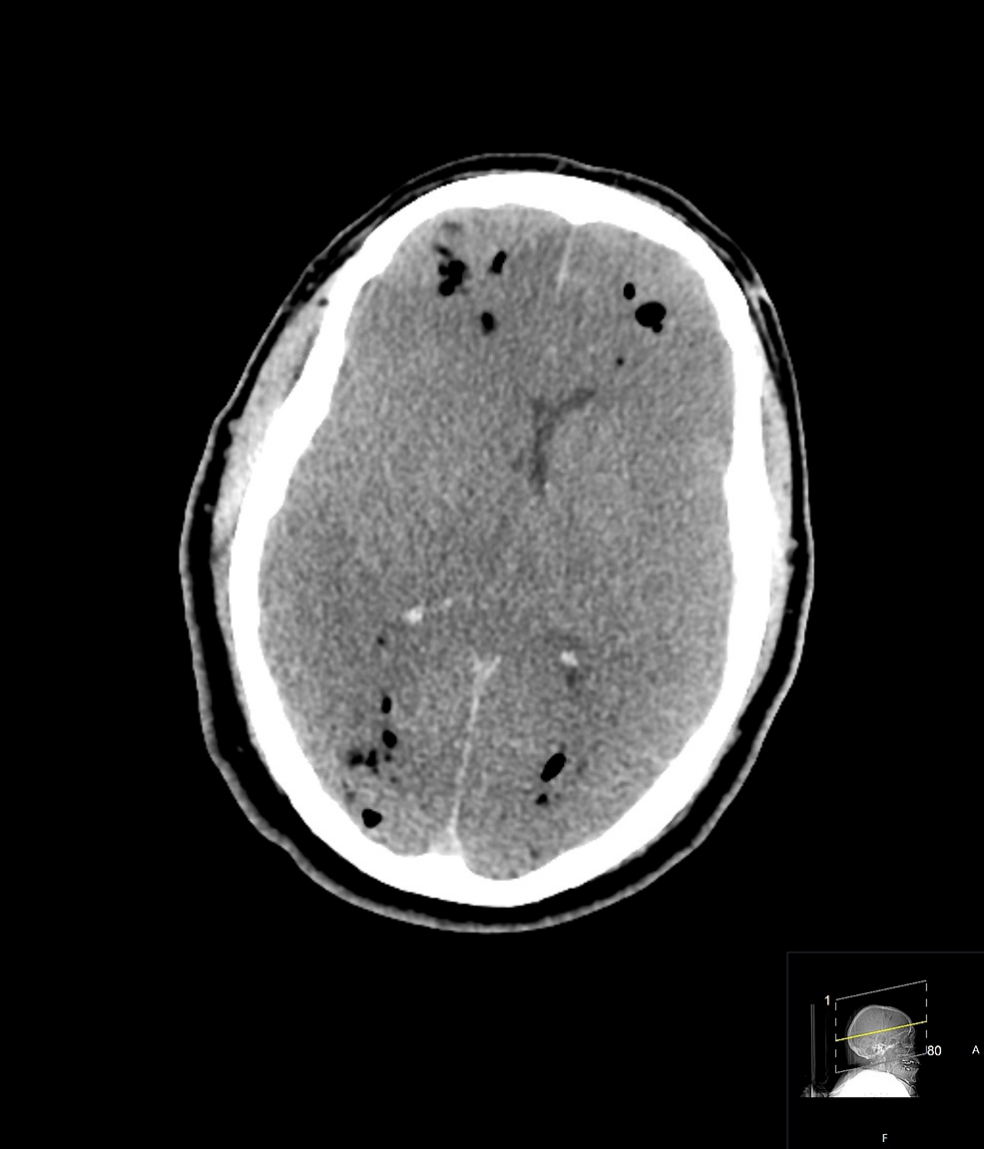
Development of extensive air scattered throughout the brain. This is likely air emboli secondary to an atrioesophageal fistula. Poor grey-white matter differentiation and loss of sulci suggesting diffuse cerebral edema, ischemia, and/or infarction. Axial brain CT without contrast. CT: computed tomography.

Imaging confirmed an atrioesophageal fistula (Figure 1). The family withdrew care after brain CT showed extensive air emboli and cerebral edema with uncal herniation (Figure 2). Air emboli were likely introduced through the fistula during the EGD.

## Discussion

Broadly, there are two techniques for ablation regarding the treatment of atrial fibrillation. The first being radiofrequency ablation, which is currently the gold standard technique at most medical centers. During radiofrequency ablation, a catheter delivers an electrical current to the point of interest. The current applies heat to the foci causing arrhythmia [3]. The second treatment is cryoablation, which utilizes a catheter to deliver a refrigerant to freeze the point of interest and inhibit any arrhythmic activity in the left atrium. The complications associated with both techniques are comparable. Major complications of ablation include esophageal fistula, thromboembolic events, and cardiac tamponade [4]. In a study of 1000 patients, the incidence of complications post-ablation was 3.9% [5]. The most common complications were tamponade, which has an incidence of 1.3%, femoral pseudoaneurysm with 0.9%, and thromboembolic events with 0.4% [5]. The incidence of atrioesophageal fistula during catheter ablation was 0.2% [5]. 

Atrioesophageal fistula is a rare but often fatal complication due to the proximity of the ectopic foci in the left atrium and pulmonary veins to the esophagus. It can present with a wide array of symptoms including fever, neurologic changes, and upper gastrointestinal bleeding. Studies have evaluated the superiority of the techniques for left atrial ablation. However, no statistically or clinically significant difference has been identified concerning efficacy and complication rate [6]. The documented procedure notes for the patient in the presented case demonstrated that when the temperature of the catheter was 0.5°C above baseline, the ablation was paused to return to baseline temperature. However, even with precautions, an atrioesophageal can still develop. Real-time esophageal temperature monitoring can assess the heat transfer to the esophagus during an ablation. However, in this case and other similar cases [7], temperature proved to be inaccurate for predicting fistula formation or heat damage to the esophagus. 

Patients with upper gastrointestinal bleeding can either be hemodynamically stable or unstable. If the patient is unstable, fluid resuscitation is needed for hypotension. Typically, if a patient is stabilized, the next step in management involves endoscopy, where air is introduced into the esophagus for better visualization [8]. However, in patients with a history of radiofrequency ablation or left atrial surgery that present with upper gastrointestinal bleeding, CT angiography of the chest and abdomen should be considered prior to EGD to look for an undiagnosed fistula. If a fistula is found on imaging, management includes prompt surgical intervention for repair of the esophageal wall [9]. Unfortunately for the presenting case, the patient had an undiagnosed atrioesophageal fistula or a thin scar tissue between the atrium and esophagus at the time of EGD. This resulted in systemic air embolism as the pressure in the esophagus increased during insufflation of air at the time of the EGD. Management of an atrioesophageal fistula should be a prompt surgical and multidisciplinary effort to prevent adverse outcomes similar to the presenting case. Survival from ablation complications such as an atrioesophageal fistula is likely correlated with high suspicion for such injury [10].

## Conclusions

The incidence of complications after catheter ablation for atrial fibrillation is very low. However, when they occur, they have high morbidity and mortality rates. This case illustrates a rare side effect of catheter ablation that was exacerbated by an EGD, causing diffuse cerebral air emboli. While patients are always consented for procedures, a more focused discussion may be warranted when an EGD is ordered for a patient with a history of heart catheterizations.
